# The heterogeneity–diversity–system performance nexus

**DOI:** 10.1093/nsr/nwad109

**Published:** 2023-04-24

**Authors:** Nico Eisenhauer, Gerrit Angst, Ana E B Asato, Rémy Beugnon, Elisabeth Bönisch, Simone Cesarz, Peter Dietrich, Stephanie D Jurburg, Anna-Maria Madaj, Rine C Reuben, Christian Ristok, Marie Sünnemann, Huimin Yi, Carlos A Guerra, Jes Hines

**Affiliations:** German Centre for Integrative Biodiversity Research (iDiv) Halle-Jena-Leipzig, Puschstr. 4, Leipzig 04103, Germany; Institute of Biology, Leipzig University, Puschstr. 4, Leipzig 04103Germany; German Centre for Integrative Biodiversity Research (iDiv) Halle-Jena-Leipzig, Puschstr. 4, Leipzig 04103, Germany; Institute of Biology, Leipzig University, Puschstr. 4, Leipzig 04103Germany; Institute of Soil Biology and Biogeochemistry, Biology Centre of the Czech Academy of Sciences, Na Sádkách 7, 37005, České Budějovice, Czech Republic; German Centre for Integrative Biodiversity Research (iDiv) Halle-Jena-Leipzig, Puschstr. 4, Leipzig 04103, Germany; Institute of Biology, Leipzig University, Puschstr. 4, Leipzig 04103Germany; German Centre for Integrative Biodiversity Research (iDiv) Halle-Jena-Leipzig, Puschstr. 4, Leipzig 04103, Germany; Leipzig Institute for Meteorology, Universität Leipzig, Stephanstraße 3, Leipzig 04103, Germany; CEFE, Univ Montpellier, CNRS, EPHE, IRD, 1919, route de Mende, F-34293 Montpellier, Cedex 5, France; German Centre for Integrative Biodiversity Research (iDiv) Halle-Jena-Leipzig, Puschstr. 4, Leipzig 04103, Germany; Institute of Biology, Leipzig University, Puschstr. 4, Leipzig 04103Germany; German Centre for Integrative Biodiversity Research (iDiv) Halle-Jena-Leipzig, Puschstr. 4, Leipzig 04103, Germany; Institute of Biology, Leipzig University, Puschstr. 4, Leipzig 04103Germany; German Centre for Integrative Biodiversity Research (iDiv) Halle-Jena-Leipzig, Puschstr. 4, Leipzig 04103, Germany; Institute of Biology, Leipzig University, Puschstr. 4, Leipzig 04103Germany; German Centre for Integrative Biodiversity Research (iDiv) Halle-Jena-Leipzig, Puschstr. 4, Leipzig 04103, Germany; Institute of Biology, Leipzig University, Puschstr. 4, Leipzig 04103Germany; Department of Environmental Microbiology, Helmholtz Centre for Environmental Research – UFZ, Leipzig 04318, Germany; German Centre for Integrative Biodiversity Research (iDiv) Halle-Jena-Leipzig, Puschstr. 4, Leipzig 04103, Germany; Institute of Biology, Leipzig University, Puschstr. 4, Leipzig 04103Germany; German Centre for Integrative Biodiversity Research (iDiv) Halle-Jena-Leipzig, Puschstr. 4, Leipzig 04103, Germany; Institute of Biology, Leipzig University, Puschstr. 4, Leipzig 04103Germany; German Centre for Integrative Biodiversity Research (iDiv) Halle-Jena-Leipzig, Puschstr. 4, Leipzig 04103, Germany; Institute of Biology, Leipzig University, Puschstr. 4, Leipzig 04103Germany; German Centre for Integrative Biodiversity Research (iDiv) Halle-Jena-Leipzig, Puschstr. 4, Leipzig 04103, Germany; Institute of Biology, Leipzig University, Puschstr. 4, Leipzig 04103Germany; German Centre for Integrative Biodiversity Research (iDiv) Halle-Jena-Leipzig, Puschstr. 4, Leipzig 04103, Germany; Institute of Biology, Leipzig University, Puschstr. 4, Leipzig 04103Germany; German Centre for Integrative Biodiversity Research (iDiv) Halle-Jena-Leipzig, Puschstr. 4, Leipzig 04103, Germany; Institute of Biology, Leipzig University, Puschstr. 4, Leipzig 04103Germany; German Centre for Integrative Biodiversity Research (iDiv) Halle-Jena-Leipzig, Puschstr. 4, Leipzig 04103, Germany; Institute of Biology, Leipzig University, Puschstr. 4, Leipzig 04103Germany

**Keywords:** heterogeneity, diversity, biodiversity-ecosystem functioning, global change, homogenization

## Abstract

Ever-growing human population and nutritional demands, supply chain disruptions, and advancing climate change have led to the realization that changes in diversity and system performance are intimately linked. Moreover, diversity and system performance depend on heterogeneity. Mitigating changes in system performance and promoting sustainable living conditions requires transformative decisions. Here, we introduce the heterogeneity–diversity–system performance (HDP) nexus as the conceptual basis upon which to formulate transformative decisions. We suggest that managing the heterogeneity of systems will best allow diversity to provide multiple benefits to people. Based on ecological theory, we pose that the HDP nexus is broadly applicable across systems, disciplines, and sectors, and should thus be considered in future decision making as a way to have a more sustainable global future.

## Teaser

This review paper suggests that managing the heterogeneity of systems will best allow diversity to provide multiple benefits to people. Based on ecological theory, we pose that the heterogeneity-diversity-performance nexus is broadly applicable across systems, disciplines, and sectors, and should thus be considered in future decision making as a way to have a more sustainable global future.

## HOMOGENIZATION IN A CHANGING WORLD

**Figure 1.**
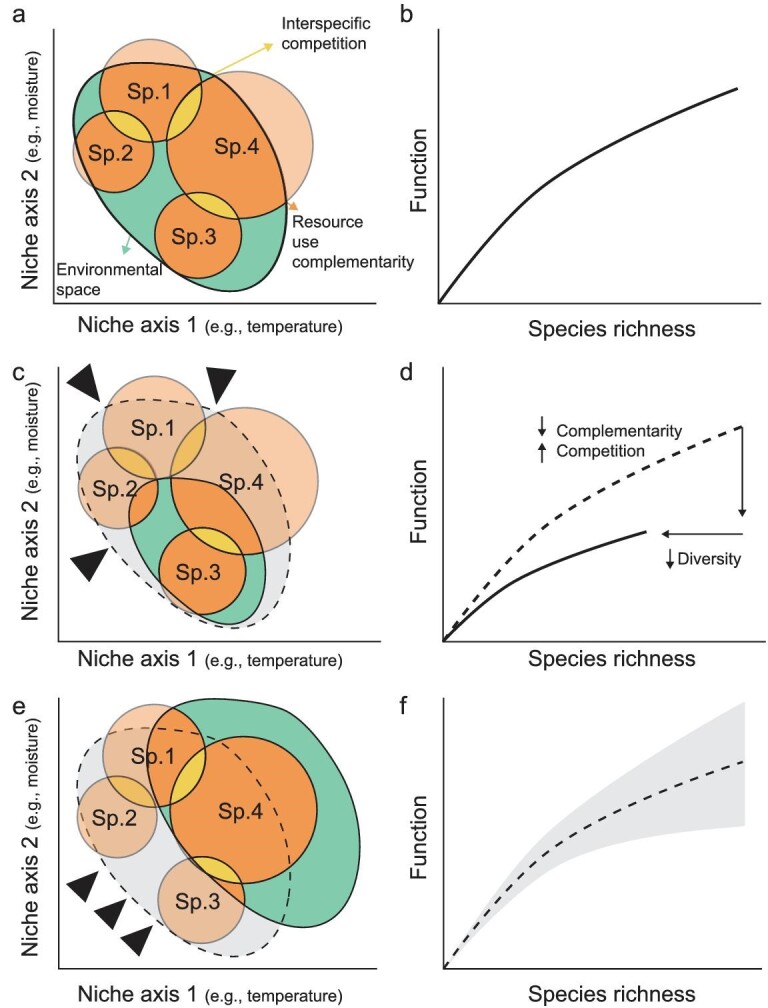
Environmental space (green) describes the environmental conditions constraining species’ coexistence and interactions, and can be described along with an infinite number of environmental parameters (a, n-dimensional hyperspace [46,47]). Environmental conditions may vary over time or in space (e.g. variable temperature or resource availability). The ecological niche (circles) is a property of the species, defined as the totality of resources and conditions necessary for its survival, growth, and reproduction [47,48]. A species’ ecological niche determines its presence, abundance, and fitness in a given environment. Non-overlapping niches support the coexistence of different species and are key to complementarity (orange) resource use and positive BEF relationships (b [14,49]). Some species may dominate communities and ecosystem functions under certain conditions, causing selection or sampling effects [14]. The degree of overlap in niches among co-occurring species (yellow) determines the presence and strength of competition among them. Environmental homogenization reduces total environmental space (c), resulting in a reduction of complementarity and a loss of biodiversity (d). In contrast, environmental shifts (e.g. due to climate change), resulting in a change, but not necessarily a reduction in the environmental space (e). While biodiversity and ecosystem function may decrease following environmental change, several mechanisms, including invasion, adaptation, and range expansion may maintain or even increase both the diversity and the functioning of these ecosystems [50] (f). Importantly, greater diversity may maintain ecosystem functioning in changing environments (i.e. the insurance hypothesis [20,21]).

**Figure 2.**
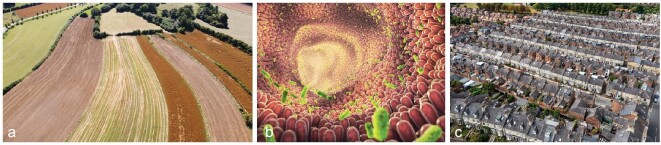
Homogenization (loss of heterogeneity) reduces diversity and influences system performance, often in unintended ways. (a) Landscape heterogeneity. Land-use change and intensification, while increasing agricultural productivity for specific crops [80], have reduced the spatial and temporal heterogeneity of environmental conditions [76], resulting in a substantial loss of biodiversity [81], ecosystem functions [82], and ecosystem services [83]. *Photo credit:* Archaecopteryx, CC BY-SA 4.0 〈https://creativecommons.org/licenses/by-sa/4.0〉, via Wikimedia Commons. (b) Gut microbiome. Similarly, industrialized human diets rely heavily on few food sources [84] that favor a select group of microbial taxa in the human gut [85] and simplified food supply chains [86]. *Photo credit:* nobeastsofierce/stock.adobe.com. (c) Urbanization. Urbanization and growing cities have also been highlighted as another main source of environmental homogenization across the globe, posing strong selective pressures on species by changing or simplifying habitat structures and environmental conditions, such as temperature, light, and pollution levels [87] as well as causing a loss of native species [88,89] and genotypic diversity [90]. Considered together, these observations indicate intimate linkages between the heterogeneity of environmental conditions, diversity of the focal system, and system performance [77]. *Photo credit:* teamjackson/stock.adobe.com.

Humans tend to homogenize the systems surrounding them in order to increase their short-term profitability and efficiency [1,2]. Homogenization, or loss of heterogeneity (Box 1), has been observed across systems (e.g. ecosystems, cities, human bodies), disciplines (e.g. ecology, economics, architecture, medicine), and scales (e.g. micro, meso, macro). However, homogenization can affect diversity and system performance in unintended ways. Here, we suggest that the underlying principles relating heterogeneity to system performance are universal and broadly applicable across disciplines. Accordingly, we introduce the general concept of the *heterogeneity–diversity–system performance nexus* (*HDP nexus)* (Box 1). This concept suggests that increases in the heterogeneity of a system can enhance the diversity of its components and, in turn, influence the performance of the system. Considering the relationships among heterogeneity, diversity, and system performance is fundamental to improving our understanding of many systems and has direct implications for the individual and collective decision making of humans. To apply the HDP nexus broadly, we define its individual components (Box 1), present basic ecological theory that supports our claims (Fig. 1; Box 2) and provide interdisciplinary examples (Fig. 2) of this phenomenon. The HDP nexus provides testable hypotheses that can be implemented across spatial and temporal scales, ranging from small scales (e.g. gut microorganisms) to whole landscapes, and across disciplines, from land management to human nutrition and health, psychology, and architecture, providing valuable insights to inform decisions that will influence system performance. Finally, we provide the example of simultaneously considering sustainable food production and consumption [3] to highlight how the HDP nexus can inform and facilitate the urgently-needed transformative changes required for a sustainable future [4,5].

Box 1. **The elements and concept of the heterogeneity–diversity–system performance nexus.**

***Heterogeneity.*** Structural or environmental variance that provides the conditions required by diversity. In ecological literature, this environmental space is often referred to as biotope space [6,7] or habitat space [8]. A large environmental space with high heterogeneity provides many niches for diversity.

***Diversity.*** Variation in the living components of a system. In ecological literature, ***biodiversity*** is the variety of life, including variation among genes, species, functional traits, functional groups, phylogenetic clades, biotic interactions, ecological networks, and ecosystems/landscapes [9]. It is often expressed as (1) richness (a measure of the number of unique life forms), (2) evenness (a measure of the equitability among life forms), and (3) β-diversity, or turnover among life forms. However, the separation of heterogeneity and diversity may not always be straightforward and needs careful consideration and definition. For instance, the diversity of plants can provide heterogeneity in structures and resources for a wide range of soil organisms [10], i.e. the biodiversity at one trophic level can beget biodiversity at other trophic levels through environmental heterogeneity [11,12].

***System performance.*** A metric quantifying the amount or extent to which an activity or process is done, i.e. an emergent process, property, or attribute that indicates the functioning of the system (e.g. productivity, stability, income). Higher levels of system performance would mean enhanced levels of productivity (e.g. in ecology, economy, or personal achievements), income (economy), success (sports) etc. The performance of ecological systems is measured as ***ecosystem functions***, which are ecological processes that control the fluxes of energy, nutrients, or organic matter through an environment [9].

**Figure:**
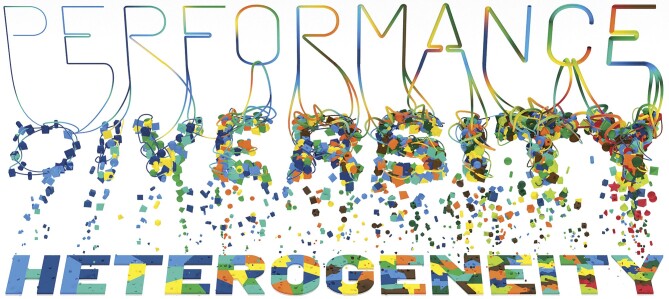


**Box 1, Figure 1. Abstract representation of the heterogeneity–diversity–system performance (HDP) nexus.** Heterogeneity is represented as the precondition for determining diversity, assuming that low heterogeneity provides few niches to support diversity. With higher levels of heterogeneity, the number and types of niches increase, as indicated by the differently colored elements of the letters in the word ‘heterogeneity’. Diversity (as represented by different geometric shapes and colors) emerges from this heterogeneity and can be described by different facets, including the number of elements, the dissimilarity of these elements (in terms of traits like shape and color [13]), and/or the interactions of these elements (depicted by the lines connecting the geometric symbols). System performance is an emergent property of this heterogeneity-driven diversity, which is mostly driven by the presence of certain geometric structures (selection or sampling effects [14]; see Fig. 1 in the main text) and the interactions among all structures in a system (complementarity effects [14]; Fig. 1). Based on these relationships, the HDP nexus describes a situation where heterogeneity begets diversity, and diversity begets system performance. Heterogeneity, diversity, and system performance increase from left to right. Although we focus on the directionality of H → D → P, there may also be feedback effects—e.g. diversity influencing the heterogeneity of a system—but considering the HDP nexus implies that humans can most effectively influence system performance by managing its heterogeneity. Based on niche theory [15–17], increasing heterogeneity should always increase diversity and performance, if it increases the number of niches for the focal diversity group. However, enhanced heterogeneity may also destroy niches, if the niche space is not large enough to support a viable population size. An example from ecology would be that a certain habitat with previously suitable conditions is fragmented too much to sustain the population of a focal species [18]. Figure in Box drawn by Gabriela Rada (iDiv).

## UNDERLYING CONCEPTS AND MECHANISMS: LEARNING FROM ECOLOGY

Coexistence and niche theory from ecology suggest that two species with the same resource requirements and without fitness differences cannot coexist on a homogenous resource in the long term, because one will always outcompete and exclude the other [15,16]. Building upon that ecological theory, the HDP nexus suggests that a heterogeneous environment is essential for diversity to persist in any complex system (Box 2, Fig. 1). Diversity, in turn, enables higher performance, i.e. the enhanced action or process of performing a task or function. For instance, when diverse components complement each other, system processes can be optimized and performance stabilized with improved resilience to disturbances [19–21]. In ecology, positive relationships between heterogeneity and biodiversity have been documented within (Fig. 1) and across (Box 2) trophic levels, i.e. biodiversity increases with increasing levels of heterogeneity. In turn, biodiversity and ecosystem function (BEF) has been observed across a variety of biological communities [9,22–24]. For example, the positive relationship between the species richness of primary producers and primary productivity has been shown across biomes [24–32]. *Niche differentiation* and *complementarity* are thought to be the main mechanisms behind positive BEF relationships (Fig. 1a,b; see also definitions and illustration of mechanisms in this figure [14,33]). Here, coexisting species fulfill different roles in an ecosystem, e.g. by using resources in dissimilar ways (different plant species may take up soil nutrients at different soil depths or points in time), thereby complementing each other and increasing community functioning (e.g. a species-rich plant community produces more biomass than low-diversity communities of the component species) [27,28,34] (Box 1). Relatedly, different species can facilitate each other by providing a more suitable abiotic and biotic environment (*facilitation effect*, which is often subsumed under the complementarity effect [33]). Moreover, the presence of well-adapted and particularly highly-productive species may contribute to positive biodiversity effects on ecosystem functioning (*selection effect*; [17]) (Box 1). Although certain plant monocultures can be highly productive, e.g. due to specific trait–environment combinations or short-term inputs of fertilizers (to compensate for nutrient depletion over time) and pesticides (to decrease the detrimental effects of accumulating pathogens over time), complementarity effects tend to dominate in the long term across settings [27,28,32], may promote win-win scenarios in agroecosystems, and may be the basis for sustainable land use [35].

Homogenization of environmental conditions that influence the *coexistence of species* are likely to affect the strength of BEF relationships [33,36,37]. Positive BEF relationships have been shown to be strongest in heterogeneous environments, and to become non-significant or even negative in homogeneous environments [8,12,38–40]. For instance, Cardinale [25] manipulated the number of algal species living in biofilms in homogeneous and heterogeneous streams. He observed that ecosystem functioning increased linearly with species richness in heterogeneous streams due to niche differences among species: different algal species dominated each unique habitat in a stream and complemented each other in driving overall ecosystem functioning [25]. This example from simple communities is supported by multiple further studies that demonstrate a strong relationship among HDP, also in more complex systems (e.g. [41–43]; Box 2).

Further, *environmental space* can be reduced through environmental homogenization, which a) decreases the number of suitable species for that environment, and b) reduces potential *complementarity* between species whilst increasing interspecific competition (Fig. 1c, d). In both cases, homogenization reduces ecosystem functioning. In the previous example, Cardinale [25] experimentally reduced the number of different niches by making all of the habitats in a stream uniform. Under these conditions, biodiversity effects on ecosystem functioning were limited and only due to the dominance of a single species (*selection effect*; Fig. 1c). Cardinale [25] concluded that communities with more species take greater advantage of the niche opportunities in an environment, resulting in elevated ecosystem functioning.

Similarly, changing environmental conditions (e.g. precipitation, climate warming, fertilization) alter the conditions that influence species coexistence. This may happen predictably (e.g. when seasonal precipitation provides a suitable environment for species [44]), or randomly (e.g. when stochastic disturbances limit habitat suitability; Fig. 1e, f). In those cases, biodiversity is also expected to stabilize ecosystem functioning by increasing the ecosystem resistance against disturbance through niche-related mechanisms, i.e. overyielding and complementarity [29,45]. In ecological systems, the existence and dominance of such biodiversity-mediated effects is associated with the degree of heterogeneity offered by the environment [21]. Niche and coexistence theory serve as a conceptual base to understand the context-dependency of BEF relationships [17,33] and diversity-performance relationships. Most importantly, if we broaden ‘biodiversity’ to ‘diversity’, and replace ‘ecosystem functioning’ by ‘performance’, the mechanisms underlying the HDP nexus could be generalized and applied across disciplines (Box 1).

Box 2. **Linkages between heterogeneity, interaction strength, diversity, and stability.**

The formal consideration of heterogeneity has played a key role in resolving a major controversy in ecology over the role of diversity in stability of food webs, one important measure of performance in [51–54]. In theory, large (i.e. diverse) networks of randomly interacting species were predicted to be unstable and prone to environmental perturbations [6,7,55]. However, this prediction contradicts observations in natural and experimental systems, where diverse networks are often more stable than species-poor ones [51,56]. This discrepancy between theoretical predictions and empirical observations caused a surge in the investigation of stabilizing features in ecological networks [57–61]. Two important determinants of network stability are the influences of heterogeneity in the *patterning* and *strength* of interactions [58,59,62–64]. That is, when patterns of species interactions are organized according to *spatial heterogeneity* in landscapes, disturbances propagate within subsystems of species that interact closely with each other within a patch, but resistance is conveyed to the landscape or system as a whole [63,64]. Examples of such heterogeneity include the stratification of water bodies [65], agricultural fields and surrounding landscapes [66], and salt marsh islands [67]. Similarly, compartmentalization has been suggested to convey stability in other types of complex systems, such as pollination [68], and banking [69]. Importantly, stability based on patterning does not need to be conveyed only by the black and white—presence and absence—of links; shades of gray also convey important components of heterogeneity. Utilizing nonlinear ecological models, McCann and colleagues [57] showed that links of weak to intermediate strength are important in promoting community persistence and stability. Theoretical work was supported by empirical results, demonstrating that some ‘weak interactors’ in food webs increased the spatiotemporal variation in community structure [58], not only highlighting the role of weak interactions in stabilizing networks but also their relationship with spatial and temporal variation [58,70].

Indeed, the classic experiment by Huffaker [71] showed that *spatial heterogeneity* could induce *stability* in predator–prey interactions and thus promote biodiversity, while spatially homogeneous conditions led to unstable dynamics and extinction. Follow-up work confirmed this finding by reporting a more stable control of population dynamics when the environment was spatially more heterogeneous and there was a balance between the extent of heterogeneity and the amount of basic food. Further support was provided by empirical [72,73] and modeling [74] work in (agricultural) landscapes of different heterogeneity. Ryser *et al.* [74] identified two main mechanisms of how landscape heterogeneity can promote biodiversity and stability under environmental change: (1) the ‘*rescue effect*’ maintains local biodiversity by rapid recolonization after a local crash in population densities; (2) the ‘*drainage effect*’ stabilizes biodiversity by preventing overshooting of population densities.

These basic principles of ecological networks may apply to a wide range of networks composed of interacting entities, including species in food webs, human or other animals transmitting infection, proteins in cells, cells in organisms (e.g. neuronal networks), gene regulatory networks, and the World Wide Web [53,75]. For instance, recent work on mammalian gene regulatory networks show that microRNAs can stabilize gene products [75]. As in the case of weak biotic interactions stabilizing food webs, weak repressions cumulatively enhanced the stability of gene regulatory networks, and broad and weak repressions conferred greater stability than a few strong ones [75]. As a consequence, we propose that heterogeneity may be a universal feature fostering weak interactions and performance across systems.

## GENERALITIES ACROSS DISCIPLINES—MOVING FROM APPLIED ECOLOGY TO OTHER FIELDS

The HDP nexus is broadly applicable to a large variety of ecological systems (e.g. [8,25,76,77]). We find rich evidence from multiple fields suggesting that the HDP nexus is also widely applicable to disciplines beyond ecology (Fig. 2). For instance, in traditional intensive farming, few crop species and varieties are planted resulting in homogenous landscapes [78], which provide few niches for multi-trophic biodiversity (e.g. herbivores and pollinators). Without high input of resources and labor, the overall performance (i.e. pollination and natural pest control) and stability of the system declines ([76]; Fig. 2a). Conversely, organic farming, intercropping, and the creation of small-scale heterogeneity can increase biodiversity in space and time, while supporting the long-term multifunctionality of ecosystems [2,76,77,79] that may be more stable to climate change and extremes [24,29].

Similarly, human diets link environmental and human health [91,92]. Globally, rising incomes and urbanization are driving dietary transitions in which traditional diets are replaced by processed diets that are rich in simple sugars, fats, but lack complex fibers [91,93]. These dietary shifts have been shown to substantially decrease the diversity and functioning of human gut microbiomes (Fig. 2b), and result in higher rates of type II diabetes, obesity, cardiovascular disease and mortality, as well as colon cancer [94]. Fiber-rich diets provide heterogeneous resources for beneficial gut microbes and can alleviate a number of conditions in their hosts, including colitis, colorectal cancer, asthma, obesity, and diabetes [95–97]. In this example, increasing the heterogeneity of fiber-rich diets enhances the biodiversity of gut microbes that drive critical body processes. This resulting elevated diversity of gut microbes thus increases human health.

Aside from the effects of dietary intake on human well-being, the heterogeneity of cityscapes also affects human physical and mental health [98]. In homogeneously-structured cities, the lack of open spaces, such as parks, recreational areas, and community hubs leads to social isolation, an increase in air pollution [99], and other health-related issues like heat stress [100]. In contrast, cities with more heterogeneous cityscapes can reduce the amount of air pollution [101], protect from global change related heat waves [102], and allow for communities to interact [103], improving overall human well-being [104].

The HDP nexus may also provide a framework to analyze human social dynamics, team composition, and success. Social media that filters information and only provides information similar to the user's viewpoints becomes homogenized over time, reducing the diversity of ideas and worldviews leading to increased vulnerability to propaganda and radicalization [105]. For instance, biases embedded in online information filtering algorithms may have unintended consequences, such as dependence on popularity signals like PageRank, trending topics, and likes, which may foster the dominance of established sources at the expense of novel ones [106,107]. Moreover, filtered news in social networks of like-minded individuals has been claimed to bias the attention of individuals toward information that they are already likely to know of or agree with [107], and resulting homogeneous social groups facilitated by online interactions may also make people more vulnerable to misinformation [107,108].

In contrast, studies have shown that communities and teams with heterogeneous backgrounds, e.g. cultural, social, and gender, support diverse ideas and approaches that may allow for holistic and inclusive problem-focused solutions [109,110]. For instance, in competitive team sports, the team with more heterogeneous skills and talents can employ more diverse tactics, increasing their chance of success [111]. Similarly, in science, multi-authored trans-disciplinary papers manage to tackle scientific questions from multiple angles, thus contributing to the advancements of multiple scientific fields simultaneously and increasing the scientific impact of the resulting research [112,113].

Although diversity is often expected to increase with increasing heterogeneity, HDP relationships may not always positively co-vary, and we might expect to see some neutral or even negative relationships between diversity and system performance as a consequence of some types of heterogeneity and in specific contexts [9]. This is expected because the benefit of heterogeneity for diversity and performance depends on a) the ability to support minimum viable population sizes, b) the complementarity of the species/elements supported, and c) on the performance of components influenced by heterogeneity (Box 1). Heterogeneity that supports complementarity among productive system components will enhance system performance and its sustainability. Based on niche theory [15,23,73], this means that increasing heterogeneity should increase diversity and performance, if it increases the number of niches for the focal diversity group (Box 1). As a consequence, high heterogeneity in a given area can also reduce the niche space for any given component, thus increasing the likelihood of stochastic extinction, ultimately reducing the overall system performance [13] and stressing the necessity to consider the appropriate spatial and temporal scale when applying the HPD nexus. Moreover, separating heterogeneity from diversity may be challenging for some examples and require a clear definition of the focal system (Box 1). However, the wealth of positive examples from ecology and beyond provides support for the utility of the HDP nexus across disciplines with important implications for decision making.

## IMPLICATIONS FOR DECISION MAKING

Ever-growing human population and nutritional demands [114], supply chain disruptions [115], advancing climate change [116], and unprecedented biodiversity loss [117], have led to the realization that changes in heterogeneity, diversity, and system performance are intimately linked [118]. Mitigating these changes and promoting sustainable living conditions requires transformative decisions [4]. The HDP nexus provides a basis upon which to formulate transformative decisions by managing the heterogeneity of systems and allowing diversity to provide multiple benefits to people. We argue that it is more promising, efficient, and straightforward to manage heterogeneity for greater diversity and meeting goals of system performance, as this approach tackles the basis of the HDP nexus and not only the outcomes. This strategy may be similar to curing a disease rather than treating its symptoms. Both the individual and collective decisions of society will benefit from the HDP nexus. For example, *individuals* that choose diverse and organic foods can not only help to reduce diseases and extend global life expectancies, but also influence the environmental effects of food production, including land clearing and greenhouse gas emissions [91]. The same principles apply to large-scale *collective* decision making by promoting the incorporation of multiple, heterogeneous values, knowledge, world views, communities, value chains, ecological concepts, and management strategies [5] and, therefore, increasing the diversity and performance of the solutions found. Having the same ecosystem types, crop varieties, species, skills, or nutrition everywhere and all the time has allowed us to simplify our lives and increase short-term productivity in unprecedented ways [119]. Notably, as known from ecology, fostering heterogeneity may not necessarily maximize individual outputs in the short term, but will enhance the sustainable supply of multiple societal and ecological benefits in the long term [79,119,120]. For instance, the HDP nexus relates to the tight connections among humans, animals, and the environment, such as acknowledged by the *One Health* concept [121]. Enhancing heterogeneity can promote diversity, which is critical to improving the health and well‐being of all components of an ecosystem [121,122]. Respective incentives need to be introduced at various political levels to encourage the multidimensional benefits of sustainably treating our bodies and ecosystems [123,124]. Currently, there are already tools to track changes in heterogeneity and their consequences for diversity and performance (c.f. essential variables [125–128]). Nevertheless, placing these tools in the context of the HDP nexus will improve support for decision making. Such knowledge about the HDP nexus and its effects on daily life and ecology needs to enter basic educational programs, such as school curricula, to enable people to make well-informed decisions affecting themselves, their fellow human beings, and future generations. In a highly connected world where humans and ecological systems are fully interdependent, the HDP nexus embraces heterogeneity across realms, which is crucial to address some of the most pressing environmental and societal challenges. In doing so, it provides a basis for transformative decisions that support global sustainability [129], and ensures that local solutions have global sustainable impacts across scales, ecosystems, issues, and sectors.
